# Praseodymium Orthoniobate and Praseodymium Substituted Lanthanum Orthoniobate: Electrical and Structural Properties

**DOI:** 10.3390/ma15062267

**Published:** 2022-03-18

**Authors:** Kacper Dzierzgowski, Sebastian Wachowski, Marcin Łapiński, Aleksandra Mielewczyk-Gryń, Maria Gazda

**Affiliations:** Institute of Nanotechnology and Materials Engineering, Faculty of Applied Physics and Mathematics, Advanced Materials Centre, Gdańsk University of Technology, Narutowicza 11/12, 80-233 Gdańsk, Poland; sebastian.wachowski@pg.edu.pl (S.W.); marcin.lapinski@pg.edu.pl (M.Ł.); alegryn@pg.edu.pl (A.M.-G.); maria.gazda@pg.edu.pl (M.G.)

**Keywords:** lanthanum orthoniobate, praseodymium orthoniobate, protonic conductivity, impedance spectroscopy, thermogravimetric analysis, thermal expansion coefficient, water uptake, triple conducting oxides

## Abstract

In this paper, the structural properties and the electrical conductivity of La_1−x_Pr_x_NbO_4+δ_ (x = 0.00, 0.05, 0.1, 0.15, 0.2, 0.3) and PrNbO_4+δ_ are presented and discussed. All synthesized samples crystallized in a monoclinic structure with similar thermal expansion coefficients. The phase transition temperature between the monoclinic and tetragonal structure increases with increasing praseodymium content from 500 °C for undoped LaNbO_4+δ_ to 700 °C for PrNbO_4+δ_. Thermogravimetry, along with X-ray photoelectron spectroscopy, confirmed a mixed 3^+^/4^+^ oxidation state of praseodymium. All studied materials, in humid air, exhibited mixed protonic, oxygen ionic and hole conductivity. The highest total conductivity was measured in dry air at 700 °C for PrNbO_4+δ_, and its value was 1.4 × 10^−3^ S/cm.

## 1. Introduction

Rare earth orthoniobates, RENbO_4_, belong to the group of materials with ABO_4_ stoichiometry. These compounds are very interesting thanks to a wide possibility of modification of their chemical composition, which allows obtaining materials with different structures and properties. The first work focused on ABO_4_ type compounds was published by Keller in 1962 [1], and in the following years, different properties of rare earth orthoniobates have been investigated. Such properties as proton conductivity [2,3,4,5,6,7,8], dielectric properties in the microwave frequency range [9,10], shape memory effect [11] and luminescence properties [12,13,14] make these materials a very promising group of materials with a wide range of applications.

Lanthanum orthoniobate was one of the ABO_4_ materials which have attracted the attention of many research groups since 2006, when Haugsrud and Norby published the work concerning proton conductivity in acceptor doped rare-earth orthoniobates [2]. Acceptor-doped LaNbO_4_ seemed to be the most promising member of this group of materials. The presented work showed that a small amount (<2%) of the acceptor, e.g., calcium, substituted for lanthanum, greatly improved the proton conductivity. This material undergoes a structural phase transition at ~500 °C that leads to a change in unit cell symmetry, proton mobility and thermal expansion coefficient (TEC) [15,16]. The development of this material was focused on the increase of total conductivity and stabilization of either the low-temperature monoclinic phase or the high-temperature tetragonal phase by applying various dopants in the lanthanum and niobium sublattice. In the lanthanum sublattice, either acceptor dopants, i.e., Ca [17], Mg [18], Sr [19], Ba [20], or isovalent dopants, i.e., Ce (III) [21], Pr (III) [6,22], Nd and Sm [23], Tb (III) [7] and Yb [24], have been applied. In order to stabilize the structure and improve the conductivity, the substitutions in the niobium sublattice such as Al, Si, P, Ga, Ge and Bi [25], Ti [26], V [27], Cr, Mn, Sb [28,29] and Ta [27], Co [30], As [31,32], Zr [33], Mo [34], Sn [35] and W [36] have been tested.

The advantages, compared to the other types of proton conducting ceramics, of the rare earth orthoniobates include high chemical stability in atmospheres containing impurities such as CO_2_ and a proton transference number close to 1. The undoubted disadvantages of rare earth niobates are low, in relation to the results obtained for perovskite materials, total conductivities and the occurrence structural transformation at temperatures around 500 °C. As a result of the transformation from the monoclinic structure to the tetragonal structure, the thermal expansion coefficient (TEC) changes by over 31% (from 12 × 10^−6^ K^−1^ to 8.3 × 10^−6^ K^−1^). Such a large change in TEC increases the probability of mechanical failure of electrochemical devices consisting of anode, electrolyte and cathode. The chosen properties of various proton-conducting materials are presented in Table 1.

The proton conductivity in the lanthanum orthoniobate has been intensively examined; however, less effort has been devoted to oxygen ionic conductivity. In the lanthanum orthoniobate, the oxygen ionic conductivity may be enhanced by introducing donor substitutions, which may lead to oxygen interstitial formation. For example, several experiments were carried out concerning the use of tungsten substitution in the niobium sublattice of LaNb_1−x_W_x_O_4+δ_ [36,52,53,54]. Another approach is to introduce a donor substitution in the lanthanum sublattice. Rare-earth cations with a mixed oxidation state seem to be suitable donor type substitutions owing to the similar ionic radii to that of lanthanum. The results obtained for cerium-doped lanthanum orthoniobate showed that, in the whole range of cerium content, a single-phase material may be obtained [21]. Additionally, some attempts to include Pr and Tb as substituents on the A-site were reported. For instance, praseodymium-doped (La_0.9_Pr_0.1_NbO_4_) [6], praseodymium and calcium co-doped (La_1−x−y_Ca_x_Pr_y_NbO_4+δ_, 0 < x < 0.02, 0 < y < 0.1) [6,22] and terbium-doped (La_1−x_Tb_x_NbO_4+δ_, 0 < x < 0.3) lanthanum orthoniobate [7] were synthesized; however, the research was focused solely on proton conductivity without consideration of oxygen ions/electron/electron-hole conductivity. Moreover, in the literature, only one work describes the conductivity of PrNbO_4+δ_ as a function of the temperature in wet air [55]. It should be noted that the properties of orthoniobates containing praseodymium are especially interesting because praseodymium can have 3^+^ and 4^+^ oxidation states.

In this work, the structural and electronic properties of praseodymium-doped lanthanum orthoniobate and praseodymium orthoniobate are presented and discussed. An improved defect chemistry model, based on the obtained results, has been presented.

## 2. Materials and Methods

Samples of La_1−x_Pr_x_NbO_4+δ_ (x = 0.00, 0.05, 0.1, 0.15, 0.2, 0.3, 1) were prepared with the use of solid-state synthesis. La_2_O_3_ (99.99% Aldrich, Saint Louis, MO, USA, preheated at 900 °C for 4 h), Pr_6_O_11_ (99.99% Aldrich, Saint Louis, MO, USA,) and Nb_2_O_5_ (99.99% Alfa Aesar, Haverhill, MA, USA) were used. The stoichiometric amounts of the oxides were hand-milled in an agate mortar with the addition of isopropanol. The obtained powders were uniaxially pressed at 400 MPa into pellets characterized with 12 mm of diameter. The green bodies were calcined at 1000 °C for 12 h. After the calcination, the specimens were ground into powders, pressed again and sintered at 1400 °C for 12 h.

The powder X-ray diffraction measurements were performed with a Philips X’Pert Pro MPD (Almelo, The Netherlands) with Cu Kα radiation. The data were analysed with the FullProf suite ((3.0, June 2015) [56]. The monoclinic (I 2/a) LaNbO_4+δ_ (ICSD: 01-073-6079) and PrNbO_4+δ_ (ICSD: 01-074-6652) structure parameters were used during the analysis [57,58]. The density of the samples was determined by an Archimedes method with kerosene as a medium.

The microstructure was characterized with FEI Quanta FEG 250 (Waltham, MA, USA) scanning electron microscope (SEM) equipped with EDAX Apollo-SD energy-dispersive X-ray spectroscopy (Mahwah, NJ, USA) (EDS) detector. The microstructure imaging was performed in High Vacuum mode with an Everhart–Thornley detector working in secondary electrons (SE) and back-scattered electrons (BSE) mode.

The praseodymium orthoniobate was characterized using X-ray photoelectron spectroscopy (XPS). The spectra were recorded with an Omicron NanoTechnology photoelectron spectrometer system (Taunusstein, Germany) operating at pressure below 1 × 10^−8^ mbar. The system incorporates a monochromatic Mg Kα X-ray source (hν = 1253.6 eV) and an Omicron Argus hemispherical analyser with a 128-channel detector. The X-ray source was operated at 20 mA emission current and 15 kV anode bias. Spectra were aligned by alignment of the C 1s core line at the binding energy of 284.8 eV. All data were analysed using the XPSPeak [59].

The dilatometry was performed by Netzsch DIL 402 PC/4 (Burlington, MA, USA) dilatometer in argon in the temperature range of 50–1000 °C with a heating rate of 3 °C/min. Thermogravimetric measurements of mass change as a function of temperature were performed in technical air and nitrogen using a Netzsch Jupiter^®^ 449 F1 (Burlington, MA, USA). The samples were heated to 900 °C with a heating/cooling rate of 2 °C/min. In the water uptake measurements, the as-prepared powders were heated to 800 °C under dry air to remove water and other impurities. The samples after dehydration were cooled to 300 °C in dry gas. After 2 h of stabilization, the dry gas was switched to the humidified gas (P_H2O_ = 0.023 atm) and, after an additional 2 h, the gas was switched back to the dry gas. Proton defect concentration was calculated with Equation (1):

(1) $$\left[OH_{O}^{•}\right]=\frac{2\cdot \Delta m\cdot M_{pr}}{m_{0}\cdot M_{H_{2}O}}$$

where Δm is a measured weight change of a sample after the switch from dry to wet gas, $M_{pr}$ denotes the molar mass of the sample, $m_{0}$ is the initial mass of the sample and $M_{H_{2}O}$ is the molar mass of water.

The electrical properties of the investigated materials were measured with impedance spectroscopy. The measurements were performed in the frequency range 1 Hz–1 MHz, with applied 1 V amplitude. Before the measurements, the samples were ink painted with platinum electrodes (ESL 5542). The measurements were performed in a wide range of oxygen (1 atm–10^−30^ atm) and water vapour (2.36 × 10^−2^ atm–6 × 10^−5^ atm) partial pressures. For the measurements performed in the air, the synthetic air mixture consisting of 20% O_2_ and 80% N_2_. The spectra were collected with Gamry Reference 3000 (Warminster, PA, USA) in a temperature range from 350 °C to 750 °C with 50 °C steps. The obtained data were analysed with ZView software. An equivalent circuit consisting of (RQ)(RQ)(RQ) elements has been used. The highest frequency semicircle is attributed to the grain interior conductivity process, the mid-frequency semicircle is related to grain boundaries and the third one, if distinguishable, describes processes related to the exchange of charge with the electrode. To identify the process for each semicircle, the capacitance was calculated with the equation:

(2) $$C=Q_{o}{}^{\frac{1}{n}}R_{n}{}^{\frac{1}{n}-1}$$

where $Q_{o}$, $R_{n}$ and *n* are pseudocapacitance, resistance and the angle of misalignment, respectively. The total conductivity ($\sigma_{\mathrm{total}}$), grain conductivity ($\sigma_{\mathrm{grains}})$ and grain boundary conductivity ($\sigma_{\mathrm{grain}\;\mathrm{boundaries}})$ were calculated, respectively, with Equations (3)–(5).

(3) $$\sigma_{\mathrm{total}}=\frac{l}{S}\;\cdot \;\frac{1}{R_{\mathrm{grains}}+R_{\mathrm{grain}\;\mathrm{boundaries}}}$$

(4) $$\sigma_{\mathrm{grains}}=\frac{l}{S}\;\cdot \;\frac{1}{R_{\mathrm{grains}}}$$

(5) $$\sigma_{\mathrm{grain}\;\mathrm{boundaries}}=\frac{l}{S}\;\cdot \;\frac{1}{R_{\mathrm{grain}\;\mathrm{boundaries}}}$$

where R denotes the resistance, l—the length of the sample, S—the area of the sample.

The Bruggeman asymmetric model was applied for porosity correction of the conductivity. The corrected value of conductivity, σ, is described with Equation (6), where $\sigma_{\exp}$ is the measured conductivity and P is the porosity fraction of the material [60].

(6) $$\sigma =\sigma_{\exp}\;\cdot \;{\left(1-P\right)}^{-\frac{3}{2}}$$

The activation energy, *E_A_*, of the conductivity was calculated with the use of the Arrhenius Equation (7):

(7) $$\sigma =\frac{\sigma_{0}}{T}\exp\left(\frac{-E_{A}}{kT}\right)$$

## 3. Results

**Figure 1:**
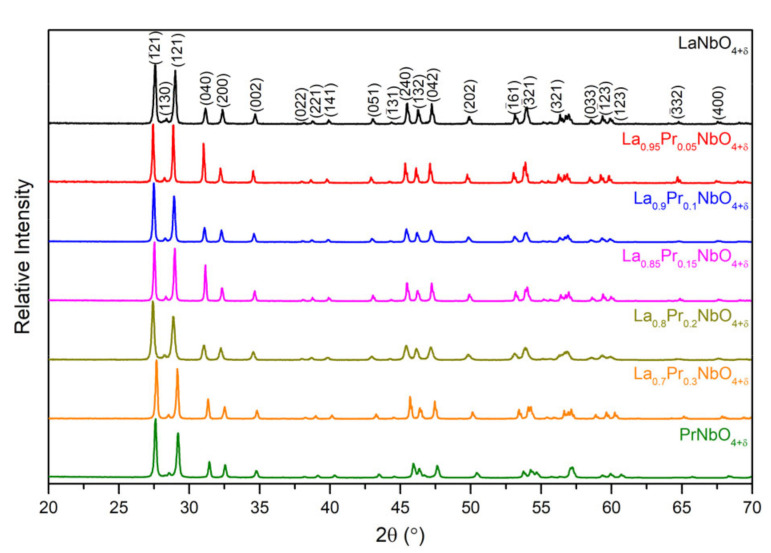
X-Ray diffractograms of the La_1−x_Pr_x_NbO_4+δ_ samples.

The XRD patterns of the La_1−x_Pr_x_NbO_4+δ_ powders are presented in Figure 1. All observed reflections for the samples with x ≤ 0.3 have been assigned to the monoclinic (*I* 2/a) LaNbO_4+δ_ structure. The pattern of praseodymium orthoniobate has been indexed with the monoclinic (I 2/a) PrNbO_4+δ_. As can be seen, all samples were obtained as single-phase materials. The XRD patterns were analysed with the LeBail refinement method. An example of the fitted profile is presented in Figure S1. The calculated structure and quality parameters are presented in Table S1.

**Figure 2:**
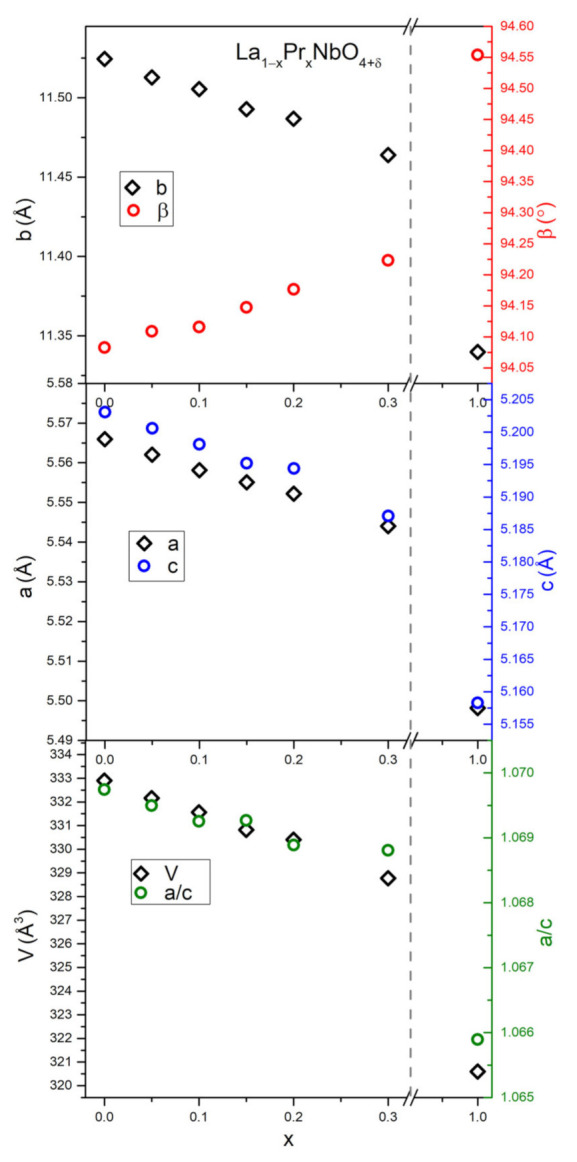
The unit cell parameters and deviation from tetragonality (a/c) of La_1−x_Pr_x_NbO_4+δ_ as a function of praseodymium content.

The calculated structure parameters (Table S1) indicate that the volume and the a, b and c unit cell parameters decrease (Figure 2), while the value of the angle β between the a and c axes increases with increasing praseodymium content. The observed changes are monotonic in the whole range of applied substitution.

**Figure 3:**
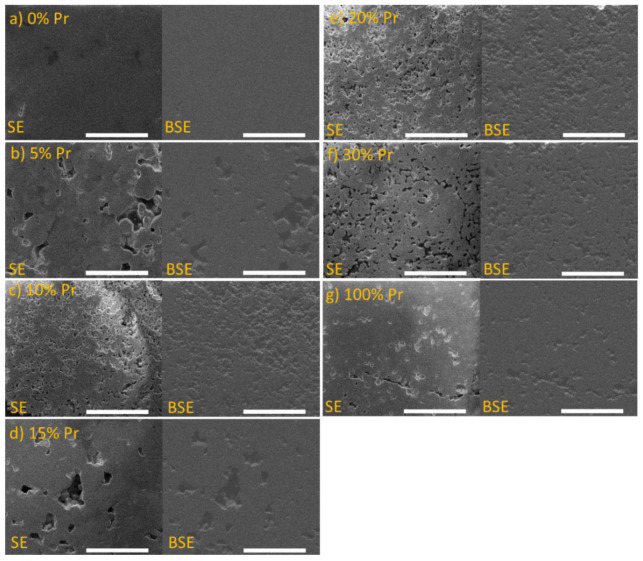
Scanning electron microscopy images of: (**a**) LaNbO_4+δ_; (**b**) La_0.95_Pr_0.05_NbO_4+δ_; (**c**) La_0.9_Pr_0.1_NbO_4+δ_; (**d**) La_0.85_Pr_0.15_NbO_4+δ_; (**e**) La_0.8_Pr_0.2_NbO_4+δ_; (**f**) La_0.7_Pr_0.3_NbO_4+δ_; (**g**) PrNbO_4+δ_ taken in SE and BSE mode. The length of the white bar corresponds to 50 µm.

Scanning electron microscopy images of polished fractures of the sintered pellets are presented in Figure 3. It can be seen that, even though each specimen was synthesized in the same conditions, the microstructures vary significantly with the Pr content. The densest pellets have been obtained for the end-members, LaNbO_4+δ_ and PrNbO_4+δ_. The ceramics of intermediate compositions show higher porosity and vary in the microstructure. The microstructure of the La_0.95_Pr_0.05_NbO_4+δ_ and La_0.85_Pr_0.15_NbO_4+δ_ sample is quite dense, and a few large pores can be seen. On the other hand, the La_0.9_Pr_0.1_NbO_4+δ_, La_0.8_Pr_0.2_NbO_4+δ_ and La_0.7_Pr_0.3_NbO_4+δ_ are characterized by numerous pores with a size below 10 μm. The analysis with EDS and BSE detectors did not show the existence of any secondary phases, which is in accordance with the XRD results. The observed differences in porosity of the samples are consistent with the results of density measurements determined by the Archimedes method, which are presented in Table 2.

**Figure 4:**
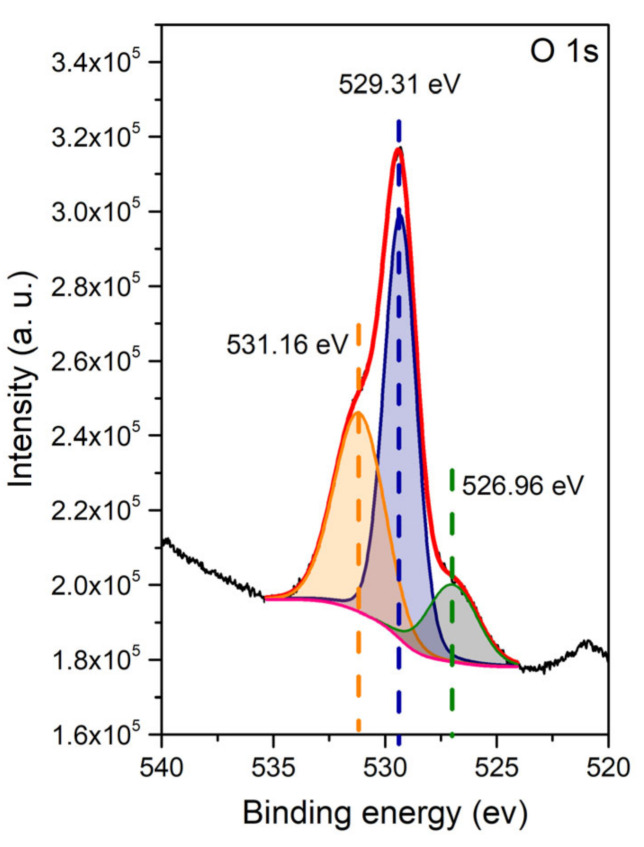
XPS spectrum collected for the O 1s band.

The XPS spectrum collected for the O 1s region is presented in Figure 4. The recorded spectrum can be deconvoluted into three peaks. The maximum at a binding energy of 529.3 eV is characteristic of the bonds of oxygen ions with niobium and praseodymium [61,62]. The maximum at 531.2 eV corresponds to O-H bonds [63]. The figure also shows the maximum at a binding energy of 527.0 eV. The presence of such a signal was also noticed by other authors in materials in which praseodymium could be in a mixed oxidation state [64,65,66,67]. In most cases, the genesis of these binding energies has not been determined. Only for SrPrGaO_4_ and PrAlO_3_ did the authors try to associate the presence of this signal with the presence of oxygen ions in interstitial positions; however, this thesis could not be confirmed in other experimental and theoretical studies [66,67]. The spectrum collected for the niobium 3d band is presented in Figure S2. Two observed maxima (209.2 and 206.4 eV) can be fitted with a single doublet with a separation energy of 2.8 eV, which is characteristic of a 5^+^ oxidation state of niobium [63,68].

**Figure 5:**
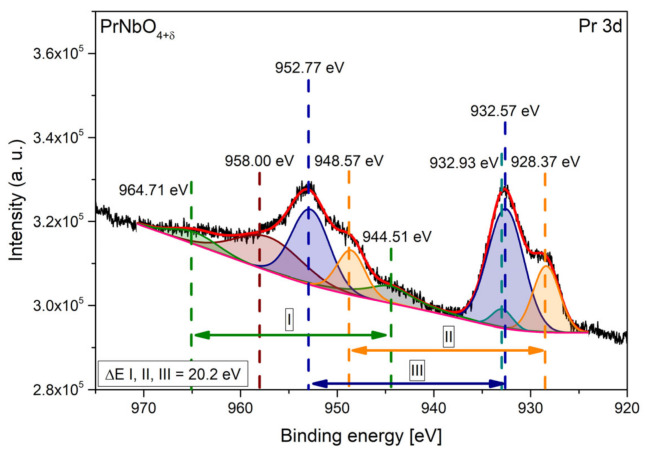
XPS spectrum collected for Pr 3d band.

The 3d praseodymium band is presented in Figure 5. It was possible to fit the spectra with three 3d_5/2–_3d_3/2_ doublets, with separation energy of 20.2 eV, and two single signals [64,69,70]. According to Schaefer et al., the maxima with binding energies of 958.0 eV and 932.9 eV are the result of observation of intra-atomic interactions [69,70]. The doublets II and III are associated with the 3d^9^4f^3^ and 3d^9^4f^2^ states, respectively, and are observed for both 4^+^ (PrO_2_) and 3^+^ (Pr_2_O_3_) praseodymium oxidation states. Doublet I represents the 3d^9^4f^1^ configuration and is observed regardless of the praseodymium oxidation state, but the intensity of this signal increases with an increase of Pr^4+^ content [69]. The 4d praseodymium band is presented in Figure S3. Some estimation of Pr(4+) may be carried out based on the XPS measurements of the Pr 4d band in the PrNbO_4+δ_. The spectra of PrNbO_4+δ_ correspond to these obtained by Lütkehoff et al. for Pr_6_O_11_ [62]. This means that the concentration of Pr(4+) is similar to the concentration of Pr(4+) in Pr_6_O_11_, which is approximately 0.66.

**Figure 6:**
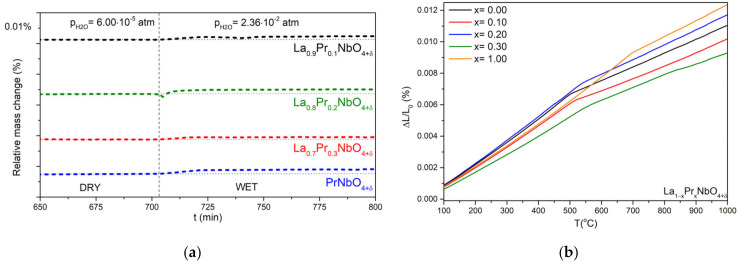
(**a**) Relative mass change observed for La_0.9_Pr_0.1_NbO_4+δ_, La_0.8_Pr_0.2_NbO_4+δ_, La_0.7_Pr_0.3_NbO_4+δ_ and PrNbO_4+δ_ at 300 °C upon a change of the atmosphere from dry to humidified air; (**b**) Relative elongation of a sample as a function of temperature for LaNbO_4+δ_, La_0.9_Pr_0.1_NbO_4+δ_, La_0.8_Pr_0.2_NbO_4+δ_, La_0.7_Pr_0.3_NbO_4+δ_ and PrNbO_4+δ_.

The thermogravimetric analysis of water uptake was performed to determine the concentration of proton defects forming in wet gases. The results obtained for La_0.9_Pr_0.1_NbO_4+δ_, La_0.8_Pr_0.2_NbO_4+δ_, La_0.7_Pr_0.3_NbO_4+δ_ and PrNbO_4+δ_ are presented in Figure 6a. The relative mass change during the gas switch was of the order of 0.002%. The calculated proton defect concentrations are shown in Table 2. The highest content of proton defects (4.9 × 10^−4^ mol/mol) was found in La_0.8_Pr_0.2_NbO_4+δ_, while the lowest was found in PrNbO_4+δ_ (2.9 × 10^−4^ mol/mol).

Dilatometry measurements were conducted to determine the phase transition temperature (T_C_) and thermal expansion coefficient (TEC). The results of the measurements are presented in Figure 6b. For all analyzed samples, two ranges, with different slopes, are observed. The observed change in the slope is related to the change in the crystal structure from low temperature monoclinic to high temperature tetragonal [71]. The TECs and values of phase transition temperature are presented in Table 3.

**Figure 7:**
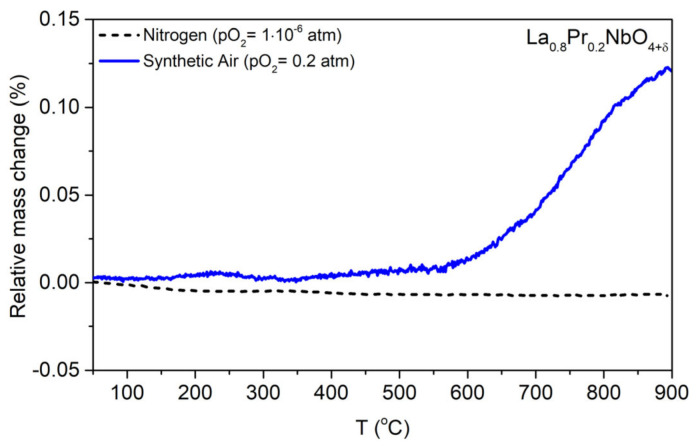
The relative mass change of La_0.8_Pr_0.2_NbO_4+δ_ as a function of temperature in air and nitrogen.

The thermogravimetric analysis (Figure 7 and Figure S4) shows that the mass of the samples changes at elevated temperatures in the air atmosphere. The result obtained for La_0.8_Pr_0.2_NbO_4+δ_, showing that in the nitrogen atmosphere the mass is constant, indirectly confirms that the increase in the mass of rare earth orthoniobates observed in the air atmosphere is related to oxygen incorporation. Measured mass changes are much smaller than the values obtained by Packer et al. for Ce_1−x_La_x_NbO_4+δ_ [21].

**Figure 8:**
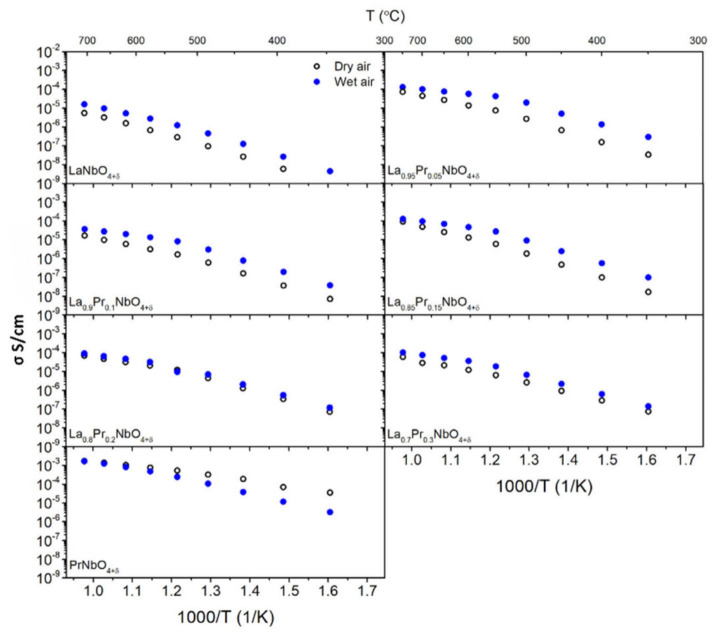
Total conductivity of La_1−x_Pr_x_NbO_4+δ_ as a function of temperature in dry (pH_2_O = 6.0 × 10^−5^ atm) and wet (pH_2_O = 2.4 × 10^−2^ atm) air.

Exemplary results of EIS measurements are presented in Figure S5. Typical values of capacitance obtained for high- and mid-frequency were 1 × 10^−11^ F/cm and 1 × 10^−10^ F/cm, respectively. The calculated conductivities of La_1−x_Pr_x_NbO_4+δ_ as a function of temperature in dry and wet air are presented in Figure 8. Table 4 summarizes the total conductivity at 400 °C and 700 °C.

**Figure 9:**
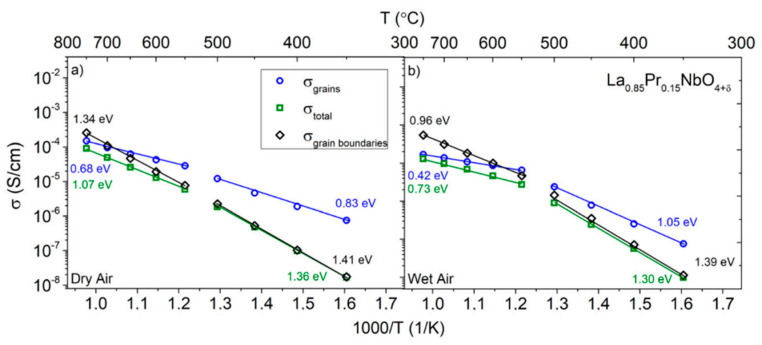
Total conductivity, grain conductivity and apparent grain boundary conductivity of La_0.85_Pr_0.15_NbO_4+δ_ as a function of temperature measured in (**a**) dry (pH_2_O = 6.0 × 10^−5^ atm) and (**b**) wet (pH_2_O = 2.4 × 10^−2^ atm) air.

The total conductivity of La_1−x_Pr_x_NbO_4+δ_ depends on the praseodymium content and the partial pressure of water vapour. In the materials with 0 ≤ x ≤ 0.3, the total conductivity in dry air was always lower than in wet air. The observed difference is the biggest at lower temperatures and decreases with increasing temperature. On the other hand, in the case of praseodymium orthoniobate, the highest total conductivities were measured in dry air. The introduction of water vapour into the air caused a decrease in conductivity. The difference between the conductivity in dry and wet air decreases with increasing temperature. All praseodymium-doped lanthanum orthoniobates are characterized by a higher total conductivity compared to lanthanum orthoniobate. The highest total conductivity (1.0 × 10^−4^ S/cm) at 700 °C in wet air was measured for La_0.95_Pr_0.05_NbO_4+δ_. The conductivity of materials with higher praseodymium content does not depend monotonically on the content of praseodymium. To analyse the influence of praseodymium content on the electrical properties of grains and grain boundaries, the components of total conductivity were determined. Figure 9 shows the conductivity of grains and grain boundaries as a function of temperature for La_0.85_Pr_0.15_NbO_4+δ_.

At temperatures higher than 650 °C in La_1−x_Pr_x_NbO_4+δ_ for 0.05 ≤ x ≤ 0.3, the conductivity of grains is lower than that of the grain boundaries. At lower temperatures, the relation between them is opposite; the conductivity of grains is higher than the conductivity of the grain boundaries. Among all studied materials, at 700 °C, La_0.9_Pr_0.1_NbO_4+δ_ and La_0.95_Pr_0.05_NbO_4+δ_ exhibit the lowest and the highest conductivity of grain boundaries, respectively (cf. Figure S6).

**Figure 10:**
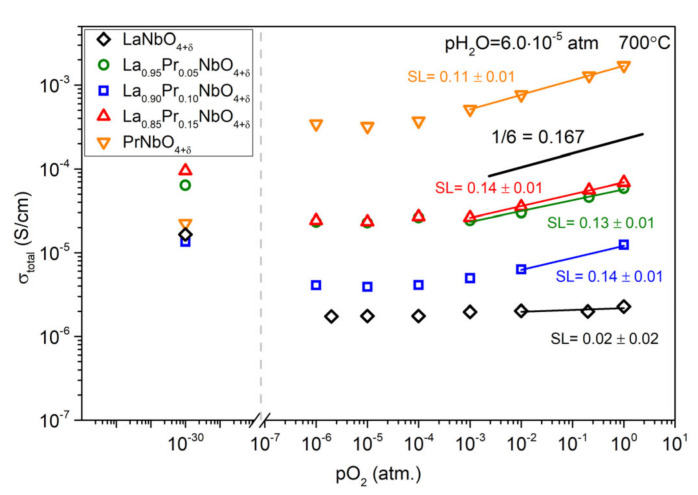
Total conductivity of La_1−x_Pr_x_NbO_4+δ_ as a function of oxygen partial pressure in dry gases (pH_2_O = 6.0 × 10^−5^ atm) at 600 °C. The SL denotes the slope coefficient of the log (σ) in a function of log (pO_2_).

**Figure 11:**
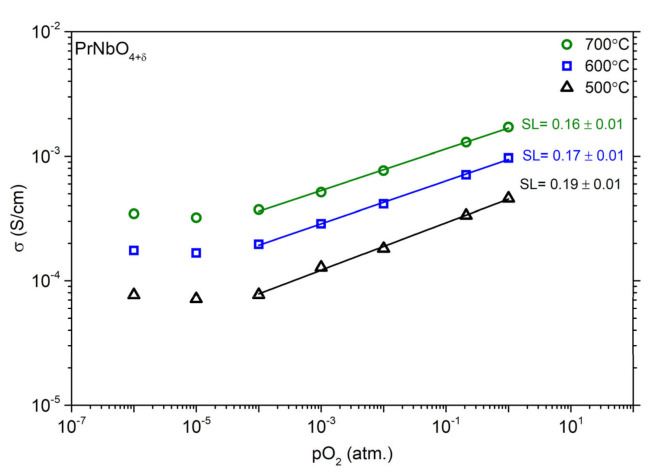
Total conductivity of PrNbO_4+δ_ as a function of oxygen partial pressure in dry gases (pH_2_O = 6.0 × 10^−5^ atm) at 500 °C, 600 °C and 700 °C. The SL denotes the slope coefficient of the log (σ) as a function of log (pO_2_).

The results of the measurements of total conductivity of La_1−x_Pr_x_NbO_4+δ_ as a function of oxygen partial pressure are presented in Figure 10. For all samples, the total conductivity decreased with decreasing oxygen partial pressure in oxidizing atmospheres and tends to flatten below 1 × 10^−3^ atm. The slope factors (SL) determined in the oxygen partial pressure range above 1 × 10^−2^ atm are positive, and for the materials containing praseodymium they are in the range of 0.13–0.16. On the other hand, for praseodymium-doped and undoped LaNbO_4+δ_, an increase in the conductivity in hydrogen (pO_2_ = 1 × 10^−30^ atm) is observed, while for PrNbO_4+δ_ a further decrease of the conductivity was observed. Measurements of total conductivity of PrNbO_4+δ_ as a function of oxygen partial pressure, at different temperatures, are presented in Figure 11. The determined values of the slope factor decrease from 0.19 to 0.16 with increasing temperature. The opposite phenomena were observed in La_0.9_Pr_0.1_NbO_4+δ_, where the slope factor value increases with increasing temperature (Figure S7).

**Figure 12:**
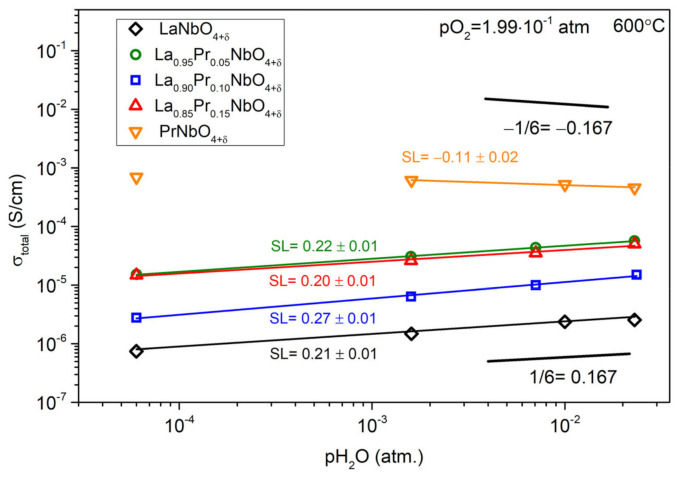
Total conductivity of La_1−x_Pr_x_NbO_4+δ_ as a function of water partial pressure in air (pO_2_ = 0.2 atm) at 600 °C. The SL denotes the slope coefficient of the line describing the log (σ) in a function of log (pH_2_O).

The results of measurements of the total conductivity as a function of the partial pressure of water vapour are presented in Figure 12. For undoped lanthanum niobate and lanthanum niobate with a praseodymium substituent, in the entire tested pH_2_O range, the total conductivity decreases with decreasing water partial pressure. In the case of PrNbO_4+δ_, the decrease of total conductivity is observed for values of pH_2_O higher than 1.0 × 10^−3^ atm.

## 4. Discussion

The observed decrease of unit cell parameters a,b,c and unit cell volume with increasing Pr content stems from the lower ionic radius of Pr^3+^ (CN = 8, r_ion_ = 1.13 Å [72]) and Pr^4+^ (CN = 8, r_ion_ = 0.96 Å [72]) compared to La^3+^ (CN = 8, r_ion_ = 1.16 Å [72]) and is in line with the results obtained for terbium-doped lanthanum orthoniobate [7]. The determined values of unit cell parameters of LaNbO_4+δ_ and PrNbO_4+δ_ are consistent with previous literature reports [73,74]. An increase of the β angle and temperature of phase transition ($T_{C}$) reflects that introducing a rare earth cation with a lower ionic radius into the lanthanum sublattice stabilizes the monoclinic structure [75]. The distance between the Pr and oxygen ion in the PrO_8_ coordination polyhedron decreases, which is associated with an increase in the binding energy. A greater binding energy means that structural changes require more energy, which in turn is associated with a higher phase transition temperature [76]. A similar phenomenon was observed in the case of lanthanum orthoniobate substituted with terbium and cerium, in which, with the increasing content of terbium or cerium in the lanthanum sublattice, an increase in the phase transition temperature was observed [7,21]. What seems unusual is the change of the a/c ratio, which decreases with increasing substituent content. Such a tendency is rather expected for dopants, which stabilize the tetragonal structure [77] because a/c, together with the β angle, reflect the deviation from tetragonality (in tetragonal structure a = c and β = 90°). The calculated values of TECs and phase transition temperatures indicate that the obtained materials, especially Pr substituted LaNbO_4_, are not suitable for wide range applications due to large changes of TEC (e.g., for LaNbO_4_ from 16.0 to 8.6) during phase transition, which occurs in the range of working temperatures of protonic devices [78,79,80].

The influence of the introduction of the rare earth element on the electrical properties of lanthanum orthoniobate is related to two factors. First, the type and content of the rare earth element may influence the concentration and/or mobility of particular types of charge carriers under the given conditions. The second factor results from the microstructure of the material, which may also depend on the content of the rare earth element. The microstructure influences the overall conductivity of the material by affecting the conductivity of the grain boundaries and the porosity of the material.

The first part of the discussion concerns the possible influence of praseodymium and its content on the charge carriers. The dependence of the total conductivity on oxygen partial pressure showed that undoped lanthanum orthoniobate in the dry atmosphere in a wide oxygen pressure range is an ionic conductor and only in oxidizing conditions, i.e., approximately pO2 ≈ 1 atm, does it become a mixed hole/oxygen ion conductor. This is in accordance with the results of computer simulations performed by Toyoura et al. [81]. The contribution of the electron-hole charge carrier to the conductivity of the materials containing praseodymium, evidenced by an increase of conductivity with increasing oxygen pressure, is observed for oxygen partial pressure higher than 10^−3^ atm. The presence of mixed conductivity in praseodymium-containing orthoniobates is related to the possible mixed valency of praseodymium. As the XPS results showed, in the analysed materials, praseodymium may occupy two oxidation levels, namely, 3+ and 4+, so that it may act either as an isovalent or a donor substituent. The change of mass of the samples recorded during thermogravimetric measurements (Figure S4) also indicates a change of the valence state of praseodymium cations.

Below, we analyse possible defect processes which may occur in both cases. The equations represent defect chemistry for La_0.8_Pr_0.2_NbO_4+δ_. Reaction (8) presents the formation of defects in La_0.8_Pr_0.2_NbO_4+δ_, with praseodymium being an isovalent (3+) substitution. In this case, oxygen nonstoichiometry, δ=0.

(8) $$0.8La_{2}O_{3}+0.2Pr_{2}O_{3}+Nb_{2}O_{5}\to 1.6La_{La}^{X}+0.4Pr_{La}^{X}+2Nb_{Nb}^{X}+8O_{O}^{X}$$

In the case of Pr^4+^, which is a donor dopant, two types of charge compensation processes may occur. The first one, which prevails in oxidizing conditions, is oxidation (Equation (9)) in which the excess positive charge of $Pr_{La}^{•}$ is compensated with the incorporation of oxygen interstitial (Equation (10)). There are also many other possibilities, where, e.g., oxidation of praseodymium may lead to formation of a cationic vacancy in the niobium sublattice; such an optional reaction has been presented in Equation (12).

(9) $$2Pr_{Pr}^{X}+\frac{1}{2}O_{2\left(g\right)}=O_{i}^{\prime \prime }+2Pr_{La}^{•}$$

(10) $$0.8La_{2}O_{3}+0.4PrO_{2}+Nb_{2}O_{5}\to 1.6La_{La}^{X}+0.4Pr_{La}^{•}+2Nb_{Nb}^{X}+8O_{O}^{X}+0.2O_{i}^{\prime \prime }$$

(11) $$0.8La_{2}O_{3}+0.4PrO_{2}+Nb_{2}O_{5}\to 1.6La_{La}^{X}+0.4Pr_{La}^{•}+2Nb_{Nb}^{X}+8O_{O}^{X}+2e^{\prime }+0.2O_{2\left(g\right)}$$

(12) $$5Pr_{La}^{X}+Nb_{Nb}^{X}+\frac{5}{2}O_{2\left(g\right)}\to 5Pr_{La}^{•}+v_{Nb}^{\prime \prime \prime \prime \prime }+\frac{1}{2}Nb_{2}O_{5}$$

The results of thermogravimetric measurements (Figure S4) suggest the incorporation of interstitial oxygen ions into the structure. The presence of oxygen interstitials was also reported for cerium and tungsten-doped lanthanum orthoniobate [54,55,81,82,83]. The second mechanism of Pr^4+^ charge compensation is the introduction of additional electrons into the conduction band (Equation (11)), which may occur in the material with a narrow energy bandgap. Another factor to consider is the location of electron-holes. Depending on thermodynamic conditions, the holes may be localized on Pr^4+^ cations, forming small polarons, or at sufficiently high temperatures they may be excited to the valence band. The latter process is described by Equation (13). In this work, it was assumed that the analysed materials are similar to other oxides containing cations on various oxidation states, such as cerium oxide doped with gadolinium and cerium vanadate, in which the dominant charge carriers are small polarons [84,85,86,87]. Therefore, in the further equations, the concentration of electron holes will be expressed by the concentration of the praseodymium 4^+^ in the lanthanum sublattice-$\left[Pr_{La}^{•}\right]$.

(13) $$Pr_{La}^{•}↔Pr_{La}^{x}+h^{•}$$

In dry atmospheres, the concentration of proton defects is negligible, and the total conductivity is the sum of hole and oxygen ion conductivities (Equation (14)):

(14) $$\sigma_{TOT}=\sigma_{Pr_{La}^{•}}+\sigma_{O}+\sigma_{OH^{•}}\approx e\mu_{Pr_{La}^{•}}\left[Pr_{La}^{•}\right]+2e\mu_{O_{i}^{\prime \prime }}\left[O_{i}^{\prime \prime }\right]$$

where the concentration of small polarons, $\left[Pr_{La}^{•}\right]$, and oxygen interstitial ions, $\left[O_{i}^{\prime \prime }\right]$, at oxidizing conditions are given as (15) and (16), respectively.

(15) $$\left[Pr_{La}^{•}\right]=K_{OX}^{\prime }{}^{\frac{1}{3}}\cdot 2^{\frac{1}{3}}\cdot p_{O_{2}}{}^{\frac{1}{6}}$$

(16) $$\left[O_{i}^{\prime \prime }\right]=K_{OX}^{\prime }{}^{\frac{1}{3}}\cdot 2^{-\frac{2}{3}}\cdot p_{O_{2}}{}^{\frac{1}{6}}$$

where $K_{OX}^{\prime }$ is a reduced constant of Reaction (9). The dependence of total conductivity on oxygen partial pressure at different temperatures (Figure S7) indicates that the concentration of tetravalent praseodymium cations changes with the temperature, which is observed in the change of the value of slope factors. These phenomena correspond to the results of thermogravimetric measurements. In the La_0.9_Pr_0.1_NbO_4+δ_, the values of the slope factor increase with the temperature from 0.11 to 0.14. The temperature range in which an SL increase is observed (500–700 °C) corresponds to that in which the change of the mass recorded with TGA, related to oxygen incorporation (Figure S4), occurs. For PrNbO_4+δ_, the behaviour is opposite. The mass of the sample during thermogravimetric measurements (Figure S4) decreases with the temperature for temperatures from 500 °C to 700 °C. This trend is also visible for the dependence of total conductivity as a function of pO_2_ for different temperatures (Figure 11), where the SL value decreases from 0.19 (for 500 °C) to 0.16 (for 700 °C).

The substitution of lanthanum with praseodymium also influences proton conductivity. As was shown by the thermogravimetric studies (Figure 6), the mass of the studied orthoniobates increases in the humidified air, which indicates the formation of proton defects. The values of the concentration of proton defects at 300 °C (2.7 × 10^−4^–4.9 × 10^−4^ mol/mol) are much lower than in classic proton conductors. For instance, the concentration of proton defects in BaZr_0.8_Y_0.2_O_3−δ_ is 4 × 10^−2^ mol/mol [88], while in La_0.98_Ca_0.02_Nb_0.9_Sb_0.1_O_4−δ_ it is 1.7 × 10^−3^ mol/mol [89]. The influence of the presence of proton defects in La_1−x_Pr_x_NbO_4+δ_ on the total conductivity (Figure 8 and Figure 11) depends on the praseodymium content. The values of the slope coefficients of the log (σ) as a function of log (pH_2_O) (0.11–0.27) indicate different mechanisms of proton conductivity compared to that of the acceptor-doped lanthanum orthoniobate and other typical proton conducting oxides. In these materials, in which the slope coefficient is around 1/2 [3,90], proton defects form with the use of oxygen vacancies. We propose that, in La_1−x_Pr_x_NbO_4+δ_, oxygen ions in interstitial positions take part in proton defect formation. It should be noted that the thermogravimetry technique does not allow us to identify whether the dominant proton defect formation reaction involves oxygen vacancy or the incorporation of oxygen into an interstitial position. On the other hand, the possibility of interstitial oxygen presence in orthoniobates containing praseodymium has already been shown. Below we analyse the processes of proton defect formation in which, together with a proton defect, an oxygen interstitial ion is formed (Equation (17)). Such a mechanism was previously analysed by Xing et al. for TiNb_2_O_7_ and Fisher et al. for Ba_2_In_2_O_5_ [91,92].

(17) $$H_{2}O_{\left(g\right)}+2O_{O}^{X}=2OH_{O}^{•}+O_{i}^{\prime \prime }$$

If this hydration reaction occurs in the atmospheres with high oxygen and water vapour partial pressure, the concentration of proton defects, small polarons, $Pr_{La}^{•}$, and oxygen interstitial ions, $O_{i}^{\prime \prime }$, are:

(18) $$\left[OH_{O}^{•}\right]=K_{OH}^{\prime }{}^{\frac{1}{3}}\cdot 2^{-\frac{1}{3}}\cdot p_{H_{2}O}{}^{\frac{1}{3}}$$

(19) $$\left[Pr_{La}^{•}\right]=K_{OX}^{\prime }{}^{\frac{1}{2}}\cdot K_{OH}^{\prime }{}^{-\frac{1}{6}}\cdot 2^{\frac{2}{3}}\cdot p_{O_{2}}{}^{\frac{1}{4}}\cdot p_{H_{2}O}{}^{-\frac{1}{6}}$$

(20) $$\left[O_{i}^{\prime \prime }\right]=K_{OH}^{\prime }{}^{\frac{1}{3}}\cdot 2^{-\frac{4}{3}}\cdot p_{H_{2}O}{}^{\frac{1}{3}}$$

where $K_{OH}^{\prime }$ is a reduced constant of Reaction (17).

As can be seen, the predicted dependencies of proton defect concentration on the water vapour partial pressure (the exponent equal to 1/3) are consistent with the values of slope coefficients (Figure 12) close to 1/3 observed in the case of La_1−x_Pr_x_NbO_4+δ_ with 0.05≤x≤0.3. This strongly suggest that, indeed, the most probable mechanism of incorporation of proton defects into the structure is based on the use of structural oxygen accompanied by interstitial oxygen formation. Moreover, these materials in wet atmospheres are mainly proton conductors, where the conductivity related to the transport of oxygen ions and polarons is lower than the proton conductivity.

In contrast to La_1−x_Pr_x_NbO_4+δ_ (0.05≤x≤0.3), the PrNbO_4+δ_, which also tends to incorporate interstitial oxygen ions, shows a different dependence of total conductivity on partial pressures of water vapour. The slope of log(σ) versus log (pH_2_O) is negative and close to −1/6. The decrease of total conductivity in wet atmospheres is also visible in the measurements as a function of temperature (Figure 8), where the total conductivity in wet air is lower than that in dry air. The observed difference between the conductivities is the highest for the lowest temperatures and decreases with an increase of the temperature. Such behaviour originates from the hydration Reaction (17), in which the incorporation of the proton defect occurs together with the incorporation of the interstitial oxygen ion. As described by Equation (19), this process leads to the decrease of concentration of small polarons $[Pr_{La}^{•}$]. The observed decrease of the total conductivity for wet air indicates that the mobile defects which dominate the total conductivity are small polarons, $[Pr_{La}^{•}$]. Therefore, proton conduction occurs in praseodymium orthoniobate; however, compared to the La_1−x_Pr_x_NbO_4+δ_ the proton component of conductivity is not dominant.

The second factor influencing the electrical properties of oxides, that is, their microstructure, cannot be neglected. The total conductivity values are affected by the porosity of the samples (Table 1). For instance, the La_0.9_Pr_0.1_NbO_4+δ_ sample, which shows total conductivity lower than these of other La_1−x_Pr_x_NbO_4+δ_ materials (Figure S6) is also the material with the lowest relative density (85%) among the synthesized materials. The comparison between this sample and La_0.95_Pr_0.05_NbO_4+δ_ shows that the grain conductivity is comparable in both materials, while the grain boundary conductivity differs by about one order of magnitude in both dry and wet atmospheres.

The introduction of the rare earth element changed the relationship between the conductivity of grains and the grain boundaries. While in the undoped lanthanum orthoniobate, at low temperatures, the conductivity of the grain boundaries was higher than that of the grains, in the samples containing praseodymium the relation was opposite; the grains were characterized by higher conductivity than that of the grain boundaries (Figure S6). Such phenomena were previously observed for terbium and calcium-doped lanthanum orthoniobate [7,19]. In our previous studies, we have shown that in the case of antimony substituted lanthanum orthoniobate, the fast transport of oxygen ions through grain boundaries takes place, so that the grain boundary conductivity is higher than the one of the grain interior [90]. In La_1−x_Pr_x_NbO_4+δ_, the higher conductivity of grain boundaries may originate from oxidation of grain boundaries, which results in a higher content of Pr^4+^ in grain boundaries than in grains. This leads to a higher concentration of holes, which exhibit higher mobility than proton defects and oxygen ions. This phenomena significantly increases the conductivity of grain boundaries compared to the conductivity of grains.

## 5. Conclusions

A series of praseodymium-substituted lanthanum orthoniobate La_1−x_Pr_x_NbO_4+δ_ (0 ≤ x ≤ 0.3) and PrNbO_4+δ_ materials were synthesized with the solid-state reaction method. All samples were single-phase, dense ceramics showing similar thermal expansion coefficients. The volume of the unit cell decreases with an increase of praseodymium content. The addition of praseodymium stabilizes the monoclinic structure, which leads to an increase of phase transition temperature from 500 °C for undoped LaNbO_4+δ_ to 700 °C for PrNbO_4+δ_. The XPS and thermogravimetric analyses indicated that praseodymium occupies the mixed 3^+^/4^+^ oxidation states.

Owing to the presence of praseodymium with a mixed oxidation state, the electrical properties of La_1−x_Pr_x_NbO_4+δ_ have changed. In atmospheres characterized with both high oxygen and water vapour partial pressures, these materials conduct three types of charges: proton defects, oxygen ions and electron-holes. Analysis of the conductivity of the materials as a function of water partial pressure indicates that the incorporation of proton defects is accompanied by oxygen interstitial formation. The highest conductivity in dry air at 700 °C was 1.4 × 10^−3^ S/cm for PrNbO_4+δ_. A non-monotonic dependence of the total conductivity on praseodymium content was observed. The influence of higher porosity and oxidation of grain boundaries on the electrical properties of materials has been discussed. In comparison to praseodymium orthoniobate, the major type of defects are proton defects, while for the PrNbO_4+δ_ it is electron holes/small polarons, $Pr_{La}^{•}$, which is commonly observed for a typical oxygen/electron hole conductor.

## Figures and Tables

**Table 1:** The basic structure of typical proton conductors under normal conditions, the total conductivity σ, the value of the TEC coefficient and the transfer number for the proton conductivity t_OH_.

Material	Structure	σ_total_ (S/cm)	TEC (10^−6^ K^−1^)	t_OH_
La_0.99_Ca_0.01_NbO_4−δ_ [17,37]	I2/c	1 × 10^−3^ (800 °C)	12 ^A^/8.3 ^B^	≈ 1
BaZr_0.8_Y_0.2_O_3−δ_ [38,39,40,41]	$Pm\bar{3}m$	2 × 10^−3^ (700 °C)	8.2	≈ 0.8
BaCe_0.8_Y_0.2_O_3−δ_ [38,42,43]	$Pm\bar{3}m$	5 × 10^−2^ (800 °C)	11.6	≈ 0.9
SrCe_0.95_Y_0.05_O_3−δ_ [16,44]	$Pm\bar{3}m$	2 × 10^−3^ (600 °C)	11.1	≈ 0.9
La_2_Zr_1.985_Ca_0.015_O_7−δ_ [45,46]	$Fd\bar{3}m$	1 × 10^−3^ (800 °C)	9.6	≈ 0.6
La_0.96_Sr_0.04_PO_4−δ_ [16,47]	$P2_{1}/c$	3 × 10^−4^ (800 °C)	10.0	≈ 1
La_0.99_Ca_0.01_TaO_4−δ_ [48,49]	$P2_{1}/c$	2 × 10^−3^ (800 °C)	5.3	≈ 1
La_0.99_Ca_0.01_VO_4−δ_ [50,51]	$P2_{1}/c$	3 × 10^−4^ (800 °C)	6.1	≈ 0.15

^A^—monoclinic structure, ^B^—tetragonal structure.

**Table 2:** Densities of sintered pellets determined with the Archimedes method. The theoretical densities are calculated with the use of the LeBail refinement results (Table S1).

Sample	Density			
	Theoretical (g/cm^3^)	Experimental (g/cm^3^)	Relative (%)	Porosity (%)
LaNbO_4+δ_	5.900	5.901	100	x
La_0.95_Pr_0.05_NbO_4+δ_	5.915	5.700	96.4	3.6
La_0.9_Pr_0.1_NbO_4+δ_	5.928	5.041	85.0	15.0
La_0.85_Pr_0.15_NbO_4+δ_	5.943	5.564	93.6	6.4
La_0.8_Pr_0.2_NbO_4+δ_	5.948	5.771	97.0	3.0
La_0.7_Pr_0.3_NbO_4+δ_	5.986	5.408	90.3	9.7
PrNbO_4+δ_	6.168	6.017	97.5	2.5

**Table 3:** Phase transition temperature (T_C_) and thermal expansion coefficient (TEC) for monoclinic and tetragonal structure and calculated proton concertation at 300 °C in wet air of La_1−x_Pr_x_NbO_4+δ_.

Sample	TEC (10^−6^/K)		T_C_ (°C)	[OH^⦁^] (10^−4^ mol/mol)
	Monoclinic	Tetragonal		
LaNbO_4_	16.0(1)	8.6(1)	500(5)	-
La_0.95_Pr_0.05_NbO_4+δ_	14.2(1)	7.5(1)	507(5)	-
La_0.9_Pr_0.1_NbO_4+δ_	14.2(1)	7.5(1)	516(4)	3.7(22)
La_0.85_Pr_0.15_NbO_4+δ_	14.6(1)	8.7(1)	526(3)	-
La_0.8_Pr_0.2_NbO_4+δ_	15.9(1)	9.2(1)	535(6)	4.9(7)
La_0.7_Pr_0.3_NbO_4+δ_	12.5(1)	8.2(1)	556(9)	2.7(7)
PrNbO_4+δ_	15.8(1)	10.0(1)	700(4)	2.9(14)

**Table 4:** The total conductivity of La_1−x_Pr_x_NbO_4+δ_ in dry (pO_2_ = 2.0 × 10^−1^ atm, pH_2_O = 6.0 × 10^−5^ atm) and wet (pO_2_ = 1.9 × 10^−1^ atm, pH_2_O = 2.4 × 10^−2^ atm) air at 450 °C and 700 °C.

	Total Conductivity (S/cm)			
Sample	700 °C		400 °C	
	Dry Air	Wet Air	Dry Air	Wet Air
LaNbO_4+δ_	3.2 × 10^−6^	9.6 × 10^−6^	5.8 × 10^−9^	5.6 × 10^−8^
La_0.95_Pr_0.05_NbO_4+δ_	4.4 × 10^−5^	1.0 × 10^−4^	1.5 × 10^−7^	1.3 × 10^−6^
La_0.9_Pr_0.1_NbO_4+δ_	1.1 × 10^−5^	3.3 × 10^−5^	8.0 × 10^−8^	5.2 × 10^−7^
La_0.85_Pr_0.15_NbO_4+δ_	4.9 × 10^−5^	9.6 × 10^−5^	9.8 × 10^−8^	5.7 × 10^−7^
La_0.8_Pr_0.2_NbO_4+δ_	4.6 × 10^−5^	6.4 × 10^−5^	3.4 × 10^−7^	5.4 × 10^−7^
La_0.7_Pr_0.3_NbO_4+δ_	2.8 × 10^−5^	7.4 × 10^−5^	2.8 × 10^−7^	6.1 × 10^−7^
PrNbO_4+δ_	1.4 × 10^−3^	1.2 × 10^−3^	7.0 × 10^−5^	1.2 × 10^−8^

## Data Availability

The data presented in this study are available on request from the corresponding author.

## Supplementary Materials

The following supporting information can be downloaded at: https://www.mdpi.com/article/10.3390/ma15062267/s1, Figure S1: The LeBail fitted profiles of the pattern and difference plot for La_0.85_Pr_0.15_NbO_4+δ_.; Figure S2: XPS spectrum of PrNbO_4+δ_ collected for Nb 3d band.; Figure S3: XPS spectrum collected for Pr 4d band, dotted lines represent the literature results measured for different praseodymium oxides by Lütkehoff et al. [48].; Figure S4: Dependence of the relative mass change of La_1−x_Pr_x_NbO_4+δ_ from temperature in the synthetic air.; Figure S5: Nyquist plot acquired for La_0.85_Pr_0.15_NbO_4+δ_ in dry and wet air at (a) 450 °C and (b) 700 °C.; Figure S6: Total conductivity, grains conductivity and grain boundaries conductivity of La_1−x_Pr_x_NbO_4+δ_ in function of praseodymium content in dry (pH_2_O = 6.0 × 10^−5^ atm) and wet (pH_2_O = 2.4 × 10^−2^ atm) air at 700 °C.; Figure S7: Total conductivity of La_0.9_Pr_0.1_NbO_4+δ_ as a function of oxygen partial pressure in dry gases (pH_2_O = 6.0 × 10^−5^ atm) at 500 °C, 600 °C and 700 °C. The SL denotes the slope coefficient of the line describing the log (σ) in a function of log (pO_2_).; Table S1: Unit cell parameters of synthetized samples of La_1−x_Pr_x_NbO_4+δ_.
