# Supplementary material for: Praseodymium Orthoniobate and Praseodymium Substituted Lanthanum Orthoniobate: Electrical and Structural Properties
Source: Materials (Basel). 2022 Mar 18;15(6):2267. doi: 10.3390/ma15062267 (PMC8954274; doi:10.3390/ma15062267)
Supplement: Supplementary file 1 [file materials-15-02267-s001.zip › materials-1564480-supplementary.pdf]

Supplementary Information

Praseodymium Orthoniobate and Praseodymium Substituted Lanthanum Orthoniobate: Electrical and Structural Properties

Kacper Dzierzgowski *, Sebastian Wachowski, Marcin Łapiński, Aleksandra Mielewczyk-Gryń and Maria Gazda

Institute of Nanotechnology and Materials Engineering, Faculty of Applied Physics and Mathematics, Advanced Materials Centre, Gdańsk University of Technology, Narutowicza 11/12, 80-233 Gdańsk, Poland; sebastian.wachowski@pg.edu.pl (S.W.); marcin.lapinski@pg.edu.pl (M.L.); alegryn@pg.edu.pl (A.M.-G.); maria.gazda@pg.edu.pl (M.G.)

* Correspondence: kacper.dzierzgowski@pg.edu.pl

Table S1. Unit cell parameters of synthetized samples of $La_{1-x}Pr_xNbO_{4+\delta}$.

Sample	a (Å)	b (Å)	c (Å)	β (°)	V (Å³)	R_{wp} (%)	χ^2
$LaNbO_{4+\delta}$	5.566(1)	11.524(1)	5.203(1)	94.083(1)	332.90(1)	9.4	4.17
$La_{0.95}Pr_{0.05}NbO_{4+\delta}$	5.562(1)	11.513(1)	5.201(1)	94.109(1)	332.16(1)	26.0	3.93
$La_{0.9}Pr_{0.1}NbO_{4+\delta}$	5.558(1)	11.505(1)	5.198(1)	94.115(1)	331.56(1)	15.4	2.13
$La_{0.85}Pr_{0.15}NbO_{4+\delta}$	5.555(1)	11.493(1)	5.195(1)	94.147(1)	330.81(1)	7.1	1.82
$La_{0.8}Pr_{0.2}NbO_{4+\delta}$	5.552(1)	11.487(1)	5.194(1)	94.176(1)	330.65(1)	6.8	2.49
$La_{0.7}Pr_{0.3}NbO_{4+\delta}$	5.544(1)	11.464(1)	5.187(1)	94.223(1)	328.77(1)	2.9	2.56
$PrNbO_{4+\delta}$	5.498(1)	11.340(1)	5.158(1)	94.554(1)	320.60(1)	6.9	2.89

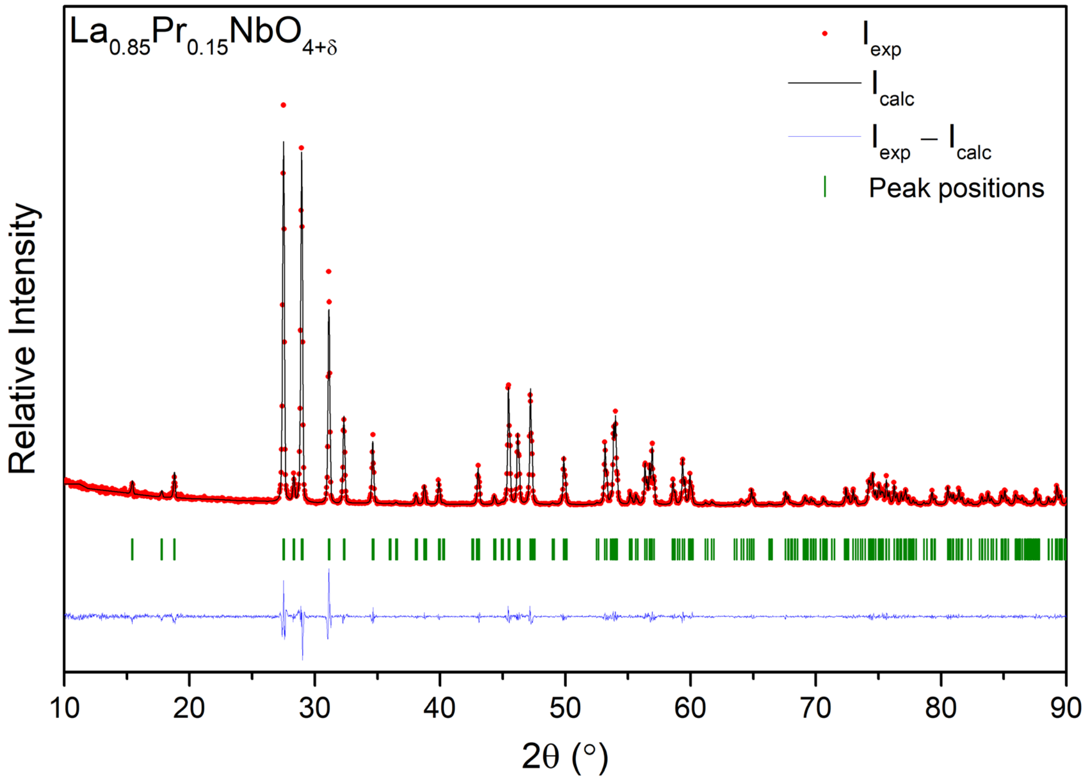

Figure S1. The LeBail fitted profiles of the pattern and difference plot for $La_{0.85}Pr_{0.15}NbO_{4+\delta}$.

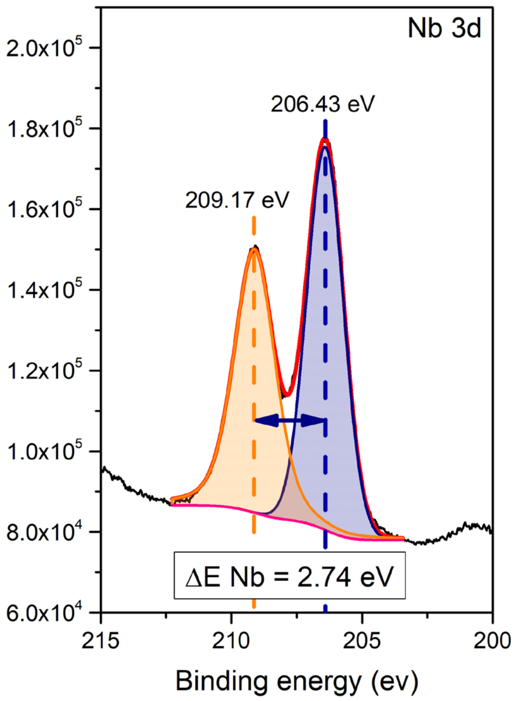

Figure S2. XPS spectrum of $PrNbO_{4+\delta}$ collected for Nb 3d band.

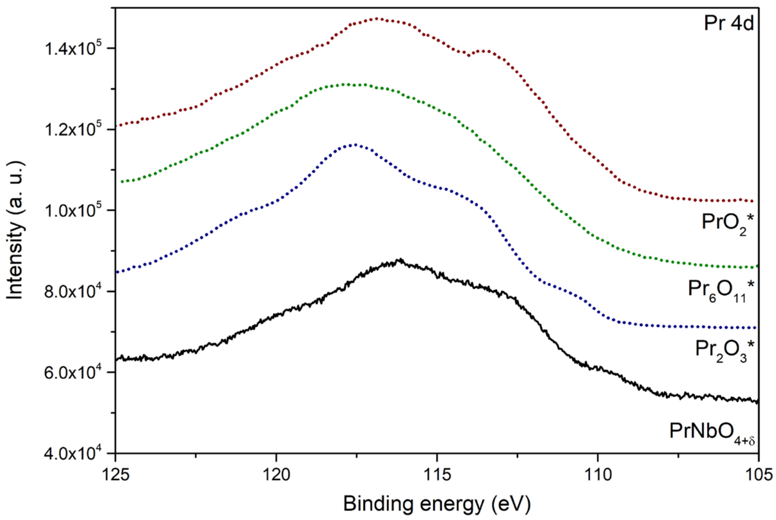

Figure S3. XPS spectrum collected for Pr 4d band, dotted lines represent the literature results measured for different praseodymium oxides by Lütkehoff et al. [65].

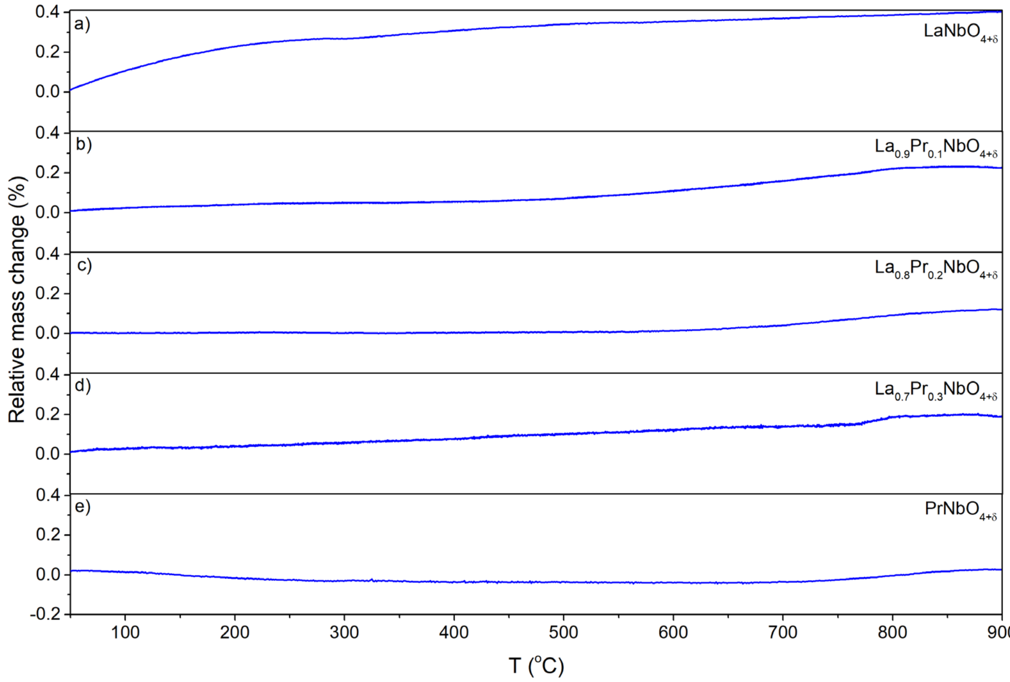

Figure S4. Dependence of the relative mass change of $La_{1-x}Pr_xNbO_{4+\delta}$ from temperature in the synthetic air.

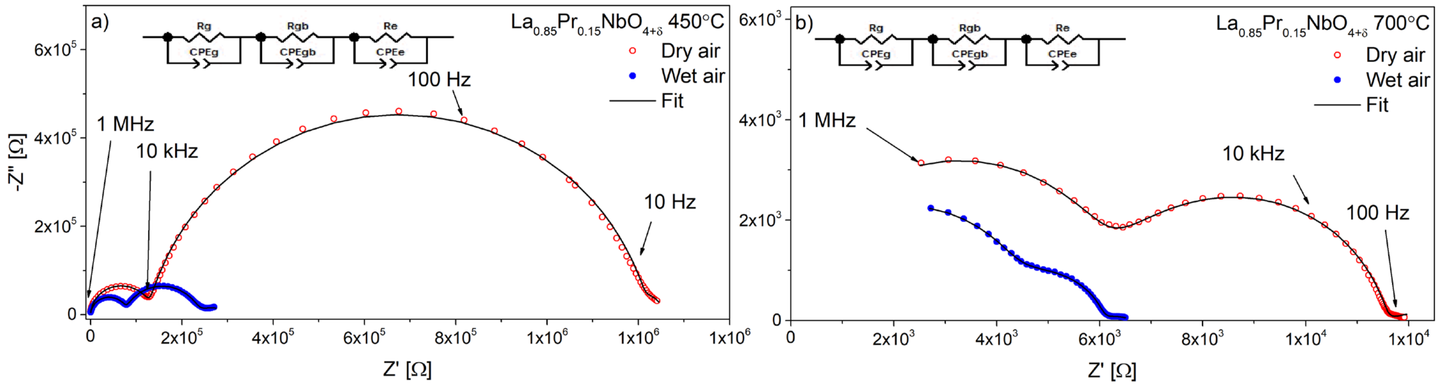

Figure S5. Nyquist plot acquired for $La_{0.85}Pr_{0.15}NbO_{4+\delta}$ in dry and wet air at a) 450°C and b) 700°C.

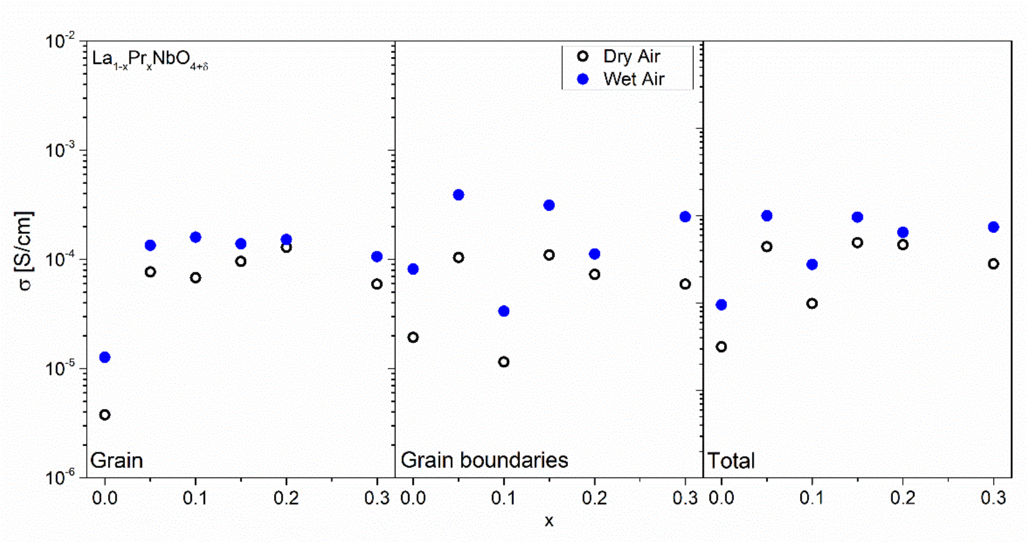

Figure S6. Total conductivity, grains conductivity and grain boundaries conductivity of $La_{1-x}Pr_xNbO_{4+\delta}$ in function of praseodymium content in dry (pH_2O= 6.0·10^{-5} atm.) and wet (pH_2O= 2.4·10^{-2} atm.) air at 700°C.

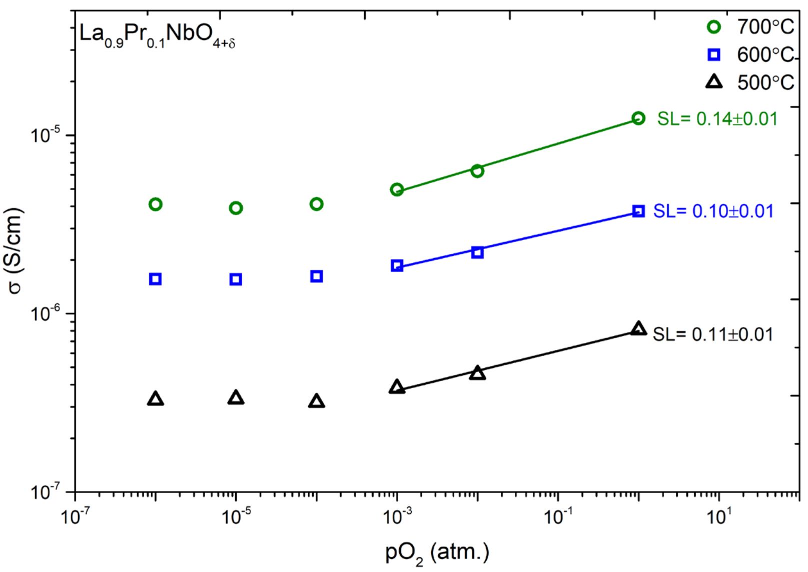

Figure S7. Total conductivity of $La_{0.9}Pr_{0.1}NbO_{4+\delta}$ as a function of oxygen partial pressure in dry gases (pH_2O= 6.0·10^{-5} atm) at 500°C, 600°C and 700°C. The SL denotes the slope coefficient of the line describing the log (σ) in a function of log (pO_2).
